# A Single Bout of Aerobic Exercise Provides an Immediate “Boost” to Cognitive Flexibility

**DOI:** 10.3389/fpsyg.2020.01106

**Published:** 2020-05-29

**Authors:** Matthew Heath, Diksha Shukla

**Affiliations:** NeuroBehavioural Laboratory, School of Kinesiology, University of Western Ontario, London, ON, Canada

**Keywords:** aerobic, executive function, exercise, oculomotor, saccade, task-switching

## Abstract

Executive function includes the core components of working memory, inhibitory control, and cognitive flexibility. A wealth of studies demonstrate that working memory and inhibitory control improve following a single bout of exercise; however, a paucity – and equivocal – body of work has demonstrated a similar benefit for cognitive flexibility. Cognitive flexibility underlies switching between different attentional- and motor-related goals, and a potential limitation of previous work examining this component in an exercise context is that they included tasks involving non-executive processes (i.e., numerosity, parity, and letter judgments). To address this issue, Experiment 1 employed a 20-min bout of aerobic exercise and examined pre- and immediate post-exercise cognitive flexibility via stimulus-driven (SD) and minimally delayed (MD) saccades ordered in an AABB task-switching paradigm. Stimulus-driven saccades are a standard task requiring a response at target onset, whereas MD saccades are a non-standard and top-down task requiring a response only *after* the target is extinguished. Work has shown that RTs for a SD saccade preceded by a MD saccade are longer than when a SD saccade is preceded by its same task-type, whereas the converse switch does not influence performance (i.e., the unidirectional switch-cost). Experiment 1 yielded a 28 ms and 8 ms unidirectional switch-cost pre- and post-exercise, respectively (*ps* < 0.001); however, the magnitude of the switch-cost was reduced post-exercise (*p* = 0.005). Experiment 2 involved a non-exercise control condition and yielded a reliable and equivalent magnitude unidirectional switch-cost at a pre- (28 ms) and post-break (26 ms) assessment (*ps* < 0.001). Accordingly, a single-bout of exercise improved task-switching efficiency and thereby provides convergent evidence that exercise provides a global benefit to the core components of executive function.

## Introduction

Executive function entails the core components of working memory, inhibitory control, and cognitive flexibility (Diamond, 2013) and is mediated via an extensive frontoparietal network (Vincent et al., 2008). Convergent evidence demonstrates that a single bout of exercise provides a short-term (<60 min) “boost” to executive function with support for this view derived largely from studies employing working memory and inhibitory control tasks (for meta analyses, see Lambourne and Tomporowski, 2010; Chang et al., 2012; Ludyga et al., 2016). For example, Li et al. (2014) used functional magnetic resonance imagery (fMRI) to examine n-back performance pre- and post- 20-min of moderate intensity aerobic exercise (via cycle ergometer at 60–70% of estimated maximal heart rate: HR_max_). The n-back task entails a string of stimuli and requires that a participant indicate when a current stimulus matches a target from n-steps earlier in the sequence (for review, see Owen et al., 2005). The task therefore provides an exemplar measure of working memory. Li et al. (2014) reported that post-exercise n-back performance was associated with a task-dependent modulation in frontoparietal activity (see also Chen et al., 2016)^1^. In turn, Chang et al. (2014) examined Stroop interference performance pre- and post- 20-min of aerobic exercise (via cycle ergometer) at a moderate intensity (65% VO_2__max_). The Stroop interference task is an exemplar measure of inhibitory control requiring that participants ignore the standard response of reporting a word name (e.g., RED) and instead provide a non-standard response of reporting the (incongruent) ink color in which the word is written (for review, see MacLeod, 1991). Chang et al. (2014) reported a 22 ms reduction in Stroop interference response times from pre- to post-exercise. Chang et al. (2014) and Li et al. (2014) attributed their findings to an exercise-based improvement in working memory and inhibitory control, respectively, with such benefits linked to: (1) increased catecholamine (Zouhal et al., 2008) and brain-derived neurotrophic factor (Knaepen et al., 2010) concentration and (2) blood flow related temperature and mechanical changes to the brain’s neural and glial networks that improve neural efficiency (i.e., the hemo-neural hypothesis) (Moore and Cao, 2008) and functional connectivity (Kelly et al., 2017).

As mentioned previously, cognitive flexibility is a core component of executive function that Diamond (2013) referred to as set-shifting and representing “…being flexible enough to adjust to changed demands or priorities” (p. 149). Cognitive flexibility builds upon inhibitory control and working memory components and emerges later in child development (Diamond, 2013). This component is most often investigated using a task-switching paradigm wherein participants alternate between different tasks in an AABB paradigm and therefore requires that participants maintain goals for two separate tasks in memory and actively inhibit one response when not specified on a current trial (Rogers and Monsell, 1995). A limited number of studies have examined whether a single bout of exercise influences task-switching and the results of this work are mixed^2^. Tsai et al. (2016) had young adults classified a low- (VO_2__max_ < 43.1 ml/kg/min) and high-fit (VO_2__max_ > 49.2 ml/kg/min) complete a 30-min single bout of exercise (via treadmill at 60% of VO_2__max_) with pre- and post-exercise task-switching efficiency assessed via alternating parity and size judgment tasks (e.g., AABB: A = parity judgment; B = size judgment). In this task, an Arabic numeral was presented at the center of a monitor and was surrounded by a solid or dashed square. When a solid square surrounded the number, participants reported if it was above or below five, whereas for a dashed square an odd/even report was provided. High-fit – but not low-fit – individuals exhibited a 58 ms post-exercise improvement in task-switching efficiency. In turn, Tomporowski and Ganio (2006) had young “recreationally active” adults complete 40-min of aerobic exercise (via cycle ergometer at 60% of VO_2__max_) and used an odd/even and vowel/consonant judgment task (AABB paradigm) to examine cognitive flexibility. In particular, when a letter-digit pair was presented in the upper quadrant of a computer monitor participants performed an odd/even judgment task, whereas when a letter-digit pair was presented in the monitor’s lower quadrant they were asked to make a consonant/vowel letter judgment. The authors reported that their exercise manipulation did not influence task-switching efficiency and concluded that the paradigm may not provide a reliable framework for measuring exercise-related changes to cognitive flexibility.

**FIGURE 1:**
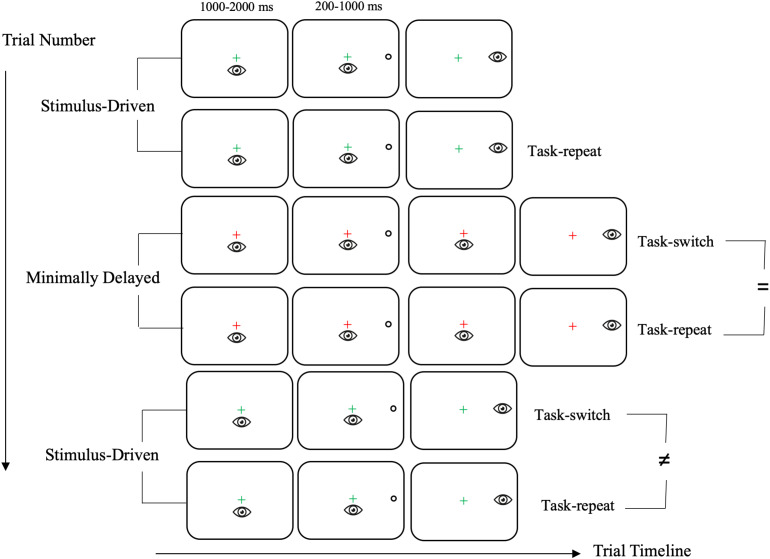
Schematic of the timeline of visual and motor events for stimulus-driven (SD) and minimally delayed (MD) task-switch and task-repeat saccades. In this schematic six consecutive trials are presented to depict an example of SD and MD task-switch and task-repeat saccades. The first trial is neither a task-switch nor a task-repeat saccade (and hence is not provided a label). The sequence shows that MD task-switch and task-repeat saccades produced equivalent RTs (i.e., see the “ = ” symbol in the schematic), whereas SD task-switch and task-repeat RTs differed (i.e., see the “≠” symbol in the schematic). In particular, SD task-switch saccades are associated with an increase in RT because trial N-1 involved a MD saccade that imparted a task-set inertia. Last, this sequence employs a green and a red fixation cross to denote SD and MD saccades respectively; however, the converse color-task mapping was also used.

The assertion that a single bout of exercise *reliably* benefits working memory and inhibitory control – but not cognitive flexibility – may be explained in the context of transcranial magnetic stimulation, lesion and neuropsychiatric studies reporting that the components of executive function are interconnected but independent processes (Thoma et al., 2007; Gilbert and Burgess, 2008). It is therefore possible that a single bout of exercise does not modulate the distinct neural circuitry underlying cognitive flexibility (for review, see Logue and Gould, 2014). Alternatively, previous work may not have used a task providing the resolution to detect *subtle* post-exercise changes in cognitive flexibility due to their inclusion of non-executive numerical magnitude, parity and letter judgment tasks – cognitive domains that do not show a reliable post-exercise benefit (Chang et al., 2012). For example, the categorical processing of Arabic numerals in a parity judgment task results in lateralized activity of the right lateral occipital area (Park et al., 2012), whereas an extensive literature has found that letter judgments result in robust activation of lateralized left temporal structures (e.g., James et al., 2005; Joseph et al., 2006); that is, the visual recognition of numbers (both magnitude and parity) and letters dissociates at both the behavioral and neural level. What is more, this behavioral dissociation is linked to the activation of non-executive cortical structures and it is known that imposing spatial constraints in an odd/even, consonant/vowel judgment task result in distinct levels of visuospatial and verbally mediated task demands (van Dijck et al., 2009). Accordingly, it is possible that task-switching paradigms that entail the non-executive domains of numerical and letter recognition do not provide the requisite resolution to reliably detect post-exercise changes in cognitive flexibility. This is a salient consideration because the additive effects of a task entailing multiple cognitive domains may preclude the ability to observe a small – but reliable – post-exercise benefit to executive function (Davranche and Audiffren, 2004). To address this issue, we had healthy young adults complete 20-min of aerobic exercise (via cycle ergometer at 80% of HR_max_) and pre- and post-exercise cognitive flexibility was examined via stimulus-driven (SD) and minimally delayed (MD) saccades performed in an AABB paradigm (i.e., A = SD saccade, B = MD saccade). The oculomotor task used here is hands-free and does not entail the perceptual categorization of a stimulus. Moreover, the task has been shown to provide the resolution to detect subtle executive changes associated with prodromal cognitive decline (Kaufman et al., 2012; Peltsch et al., 2014; Heath et al., 2017). In particular, a SD saccade is a “standard” task requiring a pre-potent goal-directed eye movement to veridical target location *at* target onset (i.e., a saccade is initiated based on exogenous target presentation). The standard nature of SD saccades is exemplified by the fact that such actions are performed upward of 150,000 times each day and are frequently referred to as reflexive in nature (Robinson, 1981). Extensive evidence has shown that SD saccades are mediated via direct retinotopic projections from the superior colliculus (Wurtz and Albano, 1980) that operate with minimal top-down executive control. In contrast, MD saccades require that participants initiate their response only *after* the target is extinguished (Figure 1). MD saccades are therefore a non-standard and endogenous task requiring top-down suppression of the standard SD saccade (i.e., do not respond at target onset) and are supported via frontoparietal executive networks (Munoz and Everling, 2004) – neural circuitry that demonstrates task-dependent changes in activity following a single bout of exercise (Hillman et al., 2011). Work by our group has shown that SD and MD saccades in an AABB paradigm result in a large magnitude (*d*_*z*_ > 1.5) *unidirectional switch-cost* (Tari et al., 2019; Tari and Heath, 2019) wherein a SD saccade preceded by a MD saccade (i.e., task-switch trial) produce a 25–30 ms increase in reaction time (RT) compared to a SD saccade preceded by its same task-type (i.e., task-repeat trial). In turn, RTs do not reliably differ for MD task-switch and task-repeat trials. Put more directly, results show that SD task-switch saccades are associated with an increase in RT compared to their task-repeat counterparts, whereas MD task-switch and task-repeat saccades do not differ (see also Figure 1). The unidirectional switch-cost has been explained via the task-set inertia hypothesis’ (Allport et al., 1994) assertion that the top-down and non-standard task-set required for a MD saccade engenders lingering neural activity that proactively delays the planning of a subsequent SD saccade. In turn, switching from a SD to MD saccade does not elicit a switch-cost because the former is implemented independent of a top-down task-set. In other words, a MD saccade requires an executive mediated task-set (or task rule) that selectively delays the planning of a subsequent and normally pre-potent SD saccade. This proposal is supported by neuroimaging (for review, see Derrfuss et al., 2005) and electroencephalographic (Heath et al., 2012; Weiler et al., 2015) evidence from humans and single-cell recording work from non-human primates (for review, see Everling and Johnston, 2013) reporting that neural activity for a non-standard task-set dissipates more slowly than a standard task.

In terms of research predictions, if a single bout of exercise improves cognitive flexibility then the magnitude, or presence, of a post-exercise unidirectional switch-cost should be reduced – a result that would provide convergent evidence that the neurobiological changes from exercise improve each core component of executive function. In turn, if exercise does not influence the unidirectional switch-cost then results would indicate that a core component of executive function (i.e., cognitive flexibility) may be immutable to a single bout of exercise. We also included a separate experiment wherein participants sat and rested on the cycle ergometer for a time equivalent to the exercise intervention. The second experiment was designed to determine whether a possible post-exercise change in task-switching efficiency was exercise-specific or related to a practice-related improvement.

## Materials and Methods

### Ethics Statement

This research was approved by the Health Sciences Research Ethics Board, University of Western Ontario, and prior to data collection all participants read a letter of information, addressed any questions to the research team, and then signed a consent form. Further, this study was conducted in accordance with latest version of the Declaration of Helsinki with the exception that participants were not registered in a database.

### Experiment 1

#### Participants

The recommended power for this type of research is 0.8 and based on our group’s previous work (Heath et al., 2018; Samani and Heath, 2018; Petrella et al., 2019; Dirk et al., 2020) it was estimated that 15–18 participants would provide statistical power of 0.8 with an alpha level of 0.5. Eighteen participants (10 female and 8 male: age range 18–25 years) from the University of Western Ontario community volunteered for this study. All participants self-reported being right-hand dominant, having normal or corrected-to-normal vision, and no current or previous history of neuropsychiatric or neurological impairment (including concussion). Participants obtained a full score on the Physical Activity Readiness Questionnaire (PAR-Q) and completed the Godin Leisure-Time Exercise Questionnaire (GLETQ; Godin, 2011). The minimum and maximum GLETQ scores were 30 and 98, respectively (Mean = 59, SD = 18) and therefore indicates that all participants were recreationally active (Amireault et al., 2015).

Participants were instructed to refrain from rigorous exercise, alcohol, and caffeine 12 h prior to the study and were encouraged to get 7 or 8 h of sleep the night before their participation. All participants reported adherence to these requests. Data collection occurred between 9:30 and 10:30 am and participants were instructed to eat a normal breakfast and to hydrate prior to the study.

#### Exercise Intervention

Participants completed a single exercise session using a height adjustable cycle ergometer (Monark 818E Ergometer, Monark Exercise AB, Vansbro, Sweden) with a heart-rate monitor affixed to their chest (Polar Wearlink + Coded Transmitter, Polar Electro Inc., Lake Success, NY, United States). The heart rate monitor was used to ensure that participants exercised within their prescribed intensity. The exercise session included a 2.5 min warm-up at 50% of participant’s HR_max_ which was followed by a step-transition to a moderate intensity (i.e., 80% of HR_max_) for 20 min. A moderate intensity was used based on our group’s work showing that a continuum of moderate to very-heavy exercise intensities does not differentially influence a post-exercise benefit to inhibitory control (Heath et al., 2018; Samani and Heath, 2018; Petrella et al., 2019; Dirk et al., 2020). During the exercise intervention, participants adjusted the resistance lever on the cycle ergometer to ensure that their heart rate was within the instructed level. Following the 20-min exercise session participants cooled down for 2.5 min at the same intensity as the warm-up interval.

#### Oculomotor Task

Prior to and following the exercise session participants sat on a height adjustable chair placed in front of a tabletop (760 mm in height) with their head secured in a head-chin rest. A 30-inch LCD monitor (60 Hz, 8 ms response rate, 1280 × 960 pixels; Dell 3007WFP, Round Rock, TX) was used to present visual stimuli and was placed at participants’ midline and 550 mm from the front edge of the table. The gaze position of participants’ left eye was measured via a video-based eye-tracking system (EyeLink 1000 Plus, SR Research, Ottawa, ON, Canada) sampling at 1000 Hz. Two additional monitors visible only to the experimenter provided real-time point of gaze information and trial-by-trial saccade kinematics (e.g., displacement, velocity). A nine-point calibration of the viewing space was performed prior to data collection and was confirmed via a follow-up validation (i.e., <1° of error for each point in the calibration grid). Computer events were controlled via MATLAB (R2018b, The MathWorks, Natick, MA, United States) and the Psychophysics Toolbox extensions (v 3.0) (Brainard, 1997; Kleiner et al., 2007) including the Eyelink Toolbox (Cornelissen et al., 2002). The lights in the experimental suite were extinguished during data collection.

Visual stimuli were presented on a black screen (0.1 cd/m^2^) and included a luminance matched central fixation cross (1.0°, 42 cd/m^2^) that appeared as green or red and open white target circles (163 cd/m^2^: 2.5° in diameter) 13.5° (i.e., proximal) and 17.5° (i.e., distal) to the left and right of the fixation cross and in the same horizontal meridian. The color of the fixation cross indicated the nature of an upcoming trial. For one half of the participants, a green fixation cross indicated a response to target location *at* target onset (i.e., stimulus-driven saccade: SD), whereas the red fixation cross indicated a response to target location *after* it was extinguished (i.e., minimally delayed saccade: MD). As a result, MD saccades required inhibition of a pre-potent response. For the other half of participants, the green and red fixation cross indicated a MD and SD saccade, respectively. Figure 1 presents the timeline of visual and motor events for SD and MD saccades. In particular, once a stable gaze was achieved (i.e., ±1.5° for 450 ms) a uniformly distributed randomized foreperiod (1000–2000 ms) was initiated after which a target was presented. The target was presented for a uniformly distributed period between 200 and 1000 ms after which it was extinguished and the fixation cross remained visible throughout a trial (i.e., overlap paradigm). Importantly, Figure 1 notes that SD saccades were initiated at target onset, whereas MD saccades were initiated only after the target was extinguished. The target presentation interval used here was based on Knox et al.’s (2018) work showing that such an interval results in longer RTs for MD than SD saccades (see also Tari et al., 2019; Tari and Heath, 2019).

Participants performed a single block of 160 trials involving SD and MD saccades alternated after every second trial (i.e., AABB: A = SD, B = MD) and were performed at pre- and post-exercise assessments. Trials were equally divided into 80 task-repeat (e.g., SD or MD saccade preceded by its same task counterpart) and 80 task-switch (e.g., SD saccade preceded by a MD saccade or vice versa) trials. The ordering of target location (i.e., left and right; proximal and distal) was pseudo-randomized in one of four pre-determined trial sequences. The first trial was counterbalanced for task-type (i.e., SD or MD) and was excluded from data analyses because it was neither a task-switch nor a task-repeat saccade. In advance of a block of trials participants were provided an instruction screen indicating that a SD saccade required that they “Look to the target as accurately as you can as soon as it appears”, whereas the instruction for a MD saccade indicated “Look at the target *only after* it disappears and complete your response as accurately as you can.” The instruction screen also indicated the fixation cross and task color mapping and specified that SD and MD saccades would be alternated after every trial. Participants were given as much time as they required to read the instruction screen and were encouraged to ask the experimenter about any questions in procedures. Further, six example trials were provided in advance of data collection.

Following the pre-exercise oculomotor assessment participants immediately started the exercise intervention. The post-exercise assessment was completed when participants’ heart rate was less than 100 beats per minute (i.e., 3–5 min following the cool-down). Each oculomotor task required 15–17 min to complete (including calibration). The timing and length of our post-exercise assessment was based on research (Chang et al., 2012; Basso and Suzuki, 2017) reporting that the largest executive benefit occurs within 20-min post-exercise.

#### Data Reduction, Dependent Variables, and Statistical Analysis

Gaze position data were filtered offline using a dual-pass Butterworth filter with a low-pass cut-off frequency of 15 Hz. Instantaneous velocity and acceleration data were computed using a five-point central-finite difference algorithm. Saccade onset was determined via velocity and acceleration values that exceeded 30°/s and 8000°/s^2^, respectively. Saccade offset was determined when velocity fell below 30°/s for 40 ms. Trials involving an eye blink (2%) were removed as were trials with a RT less than 50 ms (Wenban-Smith and Findlay, 1991) and/or an amplitude less than 2° or greater than 2.5 standard deviations beyond a participant- or condition-specific mean (Weiler et al., 2015). Less than 6% of trials were removed for the aforementioned criteria. As well, less than 5% of trials involved a task error (i.e., completing a SD instead of an instructed MD saccade and vice versa). The low error rate is attributed to the use of an overlap paradigm coupled with the predictable nature of the AABB paradigm used here and is a result directly in line with previous work (Tari et al., 2019).

**FIGURE 2:**
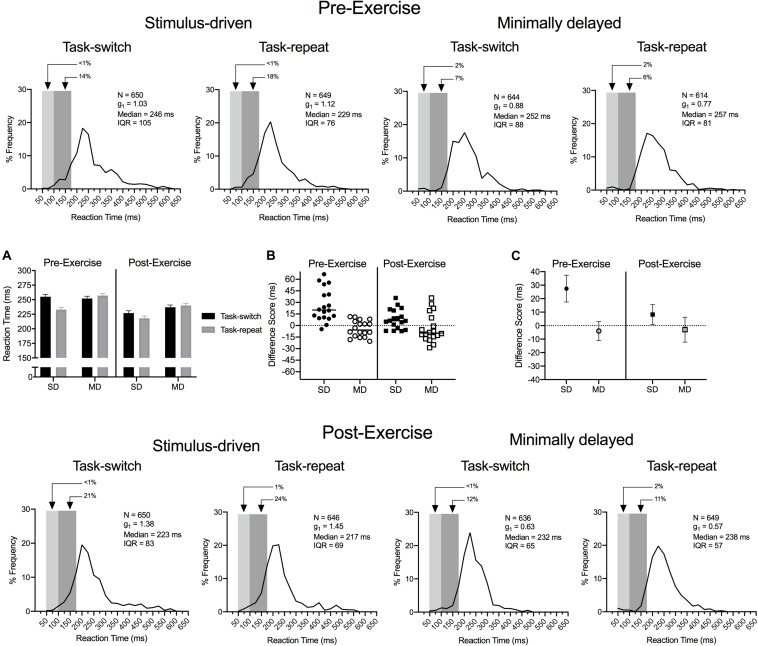
Experiment 1: The main panels show reaction time (RT) percent frequency histograms (bin width = 25 ms) for pre- and post-exercise stimulus-driven (SD) and minimally delayed (MD) task-switch and task-repeat saccades. For each panel the light and dark gray rectangles represent anticipatory (<100 ms) and short-latency (100 to <200 ms) responses, respectively. Inset panel **(A)** shows group mean RT for pre- and post-exercise task-switch and task-repeat trials with error bars representing 95% within-participant confidence intervals. Inset panels **(B,C)** represent participant-specific and group mean RT difference scores (task switch minus task-repeat), respectively. The errors bars in **(C)** represent 95% between-participant confidence intervals and the absence of overlap between an error bar and zero (i.e., horizontal dashed line) represents a reliable effect inclusive to a test of the null hypothesis.

Dependent variables included reaction time (RT: time from response cuing to saccade onset), saccade duration (time from saccade onset to saccade offset), saccade gain amplitude (i.e., saccade amplitude/veridical target amplitude) and associated within-participant standard deviations (i.e., saccade gain variability). Median RTs were computed given the positive skew of distributions (see Figure 2), whereas means were computed for saccade duration, gain and gain variability. Dependent variables were examined via 2 (assessment: pre- and post-exercise) by 2 (task: SD, MD) by 2 (task-transition: task-switch, task-repeat) fully repeated measures ANOVA. An alpha of 0.05 was set for all statistical comparisons. In addition to null hypothesis testing, two one-sided (TOST) statistics were computed to determine whether task-switch and task-repeat trials were within an equivalence boundary; that is, to conclude no difference in treatment effects (i.e., equivalence test: for tutorial see Lakens, 2017).

### Experiment 2

#### Participants

Eighteen participants (10 female and 8 male: age range 18–26 years) from University of Western Ontario community volunteered for this study and were independent of the participants recruited in Experiment 1. Experiment 2 involved the same inclusion criterion and same instruction-set (i.e., no rigorous exercise, alcohol or caffeine 12 h prior to the study) as Experiment 1. The minimum and maximum GLETQ scores were 35 and 88, respectively (Mean = 40, SD = 22) and did not reliably differ from Experiment 1 [t(34) = 0.74, *p* = 0.461].

#### Intervention and Oculomotor Assessment

The only difference with Experiment 1 was that following the initial oculomotor assessment participants sat on the cycle ergometer for 25-min (i.e., participants did not exercise) and watched a movie or tv show of their choice provided on a popular streaming application prior to completing their second oculomotor assessment. Experiment 2 was designed to determine whether repeated exposure – or practice – to the oculomotor assessment resulted in a decreased unidirectional switch-cost.

#### Data Reduction, Dependent Variables, and Statistical Analysis

Data post-processing and analyses were the same as Experiment 1. Less than 6% of trials were removed due to outlier criterion and less than 5% of trials involved a task error (i.e., an error rate comparable to Experiment 1). For the ANOVA, pre- and post-break were used instead of the pre- and post-exercise nomenclature used in Experiment 1.

## Results

### Experiment 1

#### Reaction Time

The main panels of Figure 2 show RT percent frequency histograms for pre- and post-exercise SD and MD task-switch and task-repeat saccades with anticipatory (i.e., <100 ms) and short-latency (i.e., 100 to <200 ms) responses represented via light and darker gray rectangles, respectively. The short-latency responses are of particular note because their durations are linked to response execution with minimal top-down (i.e., executive) control (Wenban-Smith and Findlay, 1991). The figure shows that SD saccades produced more short-latency responses than their MD counterparts and is a result consistent across pre- and post-exercise task-switch and task-repeat saccades. ANOVA findings revealed main effects for assessment, *F*(1,17) = 17.43, *p* < 0.001, η_p_^2^ = 0.51, task, *F*(1,17) = 5.14, *p* = 0.04, η_p_^2^ = 0.23, task-transition, *F*(1,17) = 5.62, *p* = 0.03, η_p_^2^ = 0.25, and a three-way interaction involving each variable, *F*(1,17) = 9.60, *p* < 0.01, η_p_^2^ = 0.36. Figure 2A demonstrates that RTs for SD task-switch saccades at pre- *and* post-exercise assessments were longer than their task-repeat counterparts (all *t*(17) = 7.01 and 2.69, *p* < 0.001 and 0.007). In contrast, MD task-switch and task-repeat saccades at pre- and post-exercise assessments did not reliably differ (all *t*(17) = −1.08 and −0.74, *ps* = 0.147 and 0.234), and TOST statistics indicated that the aforementioned contrasts were near – or at – a conventional level of statistical equivalence (*ts*(17) = 1.59 and 2.10, *ps* = 0.065 and 0.026).

Our *post hoc* decomposition did not uncover the nature of the time by task by task-transition interaction. Accordingly, we computed RT difference scores (task-switch minus task-repeat) for SD and MD saccades to determine whether the switch-cost magnitude was influenced by our exercise manipulation. Figure 2B presents participant-specific RT difference scores and demonstrates that SD saccades produced longer values at the pre- than post-exercise assessment. Figure 2C illustrates that the group mean SD saccade difference score was larger at the pre- than post-exercise assessment [*t*(17) = 3.18, *p* = 0.005]. In turn, MD saccade pre- and post-exercise difference scores did not reliably differ [*t*(17) = −0.55, *p* = 0.589] and were within an equivalence boundary (TOST: *t*(17) = 2.72, *p* = 0.007).

#### Movement Time, Saccade Gain and Saccade Gain Variability

**FIGURE 3:**
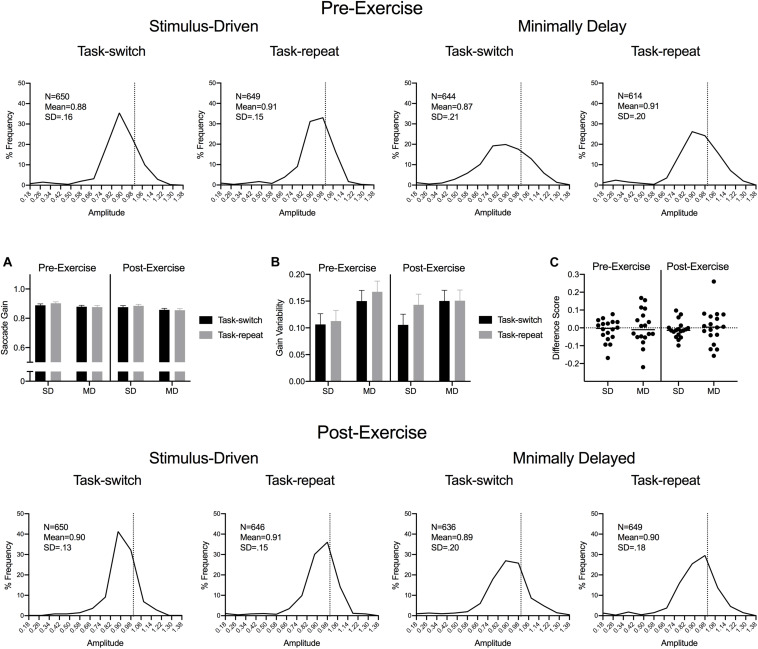
Experiment 1: The main panels show saccade gain (i.e., saccade amplitude/veridical target amplitude) percent frequency histograms (bins width = 0.10) with the vertical dashed line in each panel indicating a unitary gain. Inset panels **(A,B)** show group mean gain and gain variability for pre- and post-exercise SD and MD task-switch and task-repeat saccades with errors bars representing 95% within-participant confidence intervals. Inset panel **(C)** presents participant-specific gain difference scores (task-switch minus task-repeat).

Saccade durations were shorter for SD (55 ms, SD = 6) than MD (68 ms, SD = 10) saccades, *F*(1,17) = 100.67, *p* < 0.001, η_p_^2^ = 0.86. Figure 3 presents saccade gain percent frequency histograms for SD and MD task-switch and task-repeat trials at pre- and post-exercise assessments. The histograms demonstrate the well-documented saccade undershooting bias (Harris, 1995) and indicates that values did not vary across the different experimental conditions used here. ANOVA results did not reveal significant main effects or interactions, all *F*(1,17) < 1.19, *ps* > 0.289, all η_p_^2^ < 0.07. Saccade gain variability yielded a main effect of task, *F*(1,17) = 17.47, *p* = 0.001, η_p_^2^ = 0.51: values were less for SD (0.11, SD = 0.05) than MD saccades (0.15, SD = 0.05) (Figure 3C).

### Experiment 2

#### Reaction Time

**FIGURE 4:**
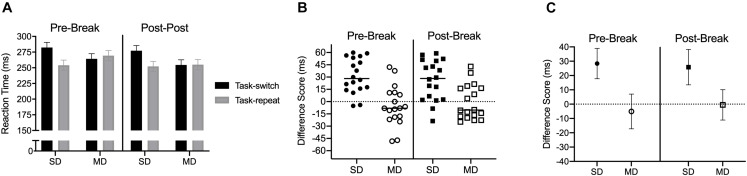
Experiment 2: Panel **(A)** presents group mean reaction time (RT) for pre- and post-break stimulus-driven and minimally delayed task-switch and task-repeat saccades with errors bars representing 95% within-participant difference scores. Panels **(B,C)** present participant-specific and group mean RT difference scores (task-switch minus task-repeat), respectively. The errors bars in **(C)** represent 95% between-participant confidence intervals.

Results produced a main effect of task-transition, *F*(1,17) = 20.72, *p* < 0.001, η_p_^2^ = 0.55, and a task by task-transition interaction, *F*(1,17) = 19.58, *p* < 0.001. η_p_^2^ = 0.53. Figure 4A shows that RTs for SD task-switch trials were longer than task-repeat trials [*t*(17) = 5.81, *p* < 0.001], whereas MD task-switch and task-repeat trials did not reliably differ [*t*(17) = −0.77, *p* = 0.449] and the TOST statistic indicated that MD task-switch and task-repeat trials approached a conventional equivalence boundary [*t*(17) = 1.98, *p* = 0.064]. Figure 4B shows participant-specific difference scores and demonstrates a SD – but not MD – switch-cost at pre- and post-break assessments. Figure 4C shows that the magnitude of the SD switch-cost did not vary across the two assessments [*t*(17) = 0.94, *p* = 0.36], and the TOST statistic indicated that SD switch-costs were within an equivalence boundary [*t*(17) = 2.07, *p* = 0.027].

#### Movement Time, Saccade Gain and Saccade Gain Variability

**FIGURE 5:**
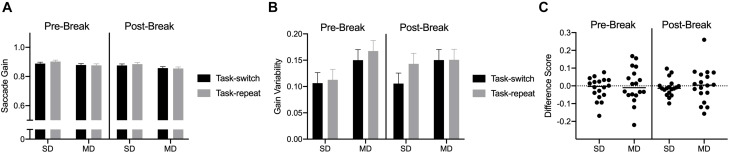
Experiment 2: Panels **(A,B)** show group mean gain and gain variability for pre- and post-break SD and MD task-switch and task-repeat saccades with errors bars representing 95% within-participant confidence intervals. Panel **(C)** presents participant-specific gain difference scores (task-switch minus task-repeat).

Saccade durations revealed a main effect of task, *F*(1,17) = 77.69, η_p_^2^ = 0.82: values were shorter for SD (54 ms, SD = 4) than MD (68 ms, SD = 9) saccades. Saccade gain did not reveal any reliable effects or interactions, all *F*(1,17) < 2.06, *p*s > 0.16, all η_p_^2^ < 0.11 (Figure 5A). Saccade gain variability indicated that values were less for SD (0.11, SD = 0.05) than MD (0.15, SD = 0.06) saccades, *F*(1,17) = 17.45, *p* = 0.001, η_p_^2^ = 0.50 (see Figures 5B,C).

## Discussion

Experiment 1 examined whether 20 min of moderate intensity aerobic exercise improves cognitive flexibility. To address this, SD and MD saccades were ordered in an AABB paradigm to examine task-switching efficiency at pre- and post-exercise assessments. Experiment 2 addressed whether a putative post-exercise improvement in task-switching efficiency is exercise-specific or relates to a practice-related improvement in the oculomotor task used here.

### Experiment 1: A Task-Set Inertia Underlies a Pre-exercise Unidirectional Switch-Cost

Wolohan and Knox (2014) and Knox et al. (2018) examined SD and MD saccades in separate blocks and reported longer RTs in the latter condition across a 200–1000 ms target delay interval (i.e., the intervals used here). Based on these findings, the authors proposed that MD saccades provide a direct measure of executive control that is less influenced by “additional” cognitive processes (i.e., attention, working memory) than the more frequently used antisaccade task. Additionally, previous work by our group showed that SD and MD saccades ordered in an AABB paradigm result in a unidirectional switch-cost. In particular, Tari et al. (2019) reported that RTs for SD task-switch saccades were on average 30 ms longer than their task-repeat counterparts, whereas RTs for MD task-switch and task-repeat trials did not reliably differ (see also Tari and Heath, 2019). In line with previous work, the pre-exercise findings of Experiment 1 yielded a 28 ms (CI_95__%_ = 9) increase in RTs for SD task-switch compared to task-repeat saccades and this contrast was associated with a large effect size (*d*_z_ = 1.65). In turn, RTs for pre-exercise MD task-switch and task-repeat saccades did not reliably differ and a TOST statistic indicated that this comparison approached (but did not attain) a conventional level of statistical equivalence (*p* = 0.065). Accordingly, the pre-exercise RT findings indicate a reliable unidirectional switch-cost and is a result taken to evince that the top-down task-set necessary to produce a non-standard MD saccade engenders lingering neural activity that proactively delays the planning of a subsequent SD saccade (i.e., the task-set inertia hypothesis) (Allport et al., 1994; Weiler et al., 2015).

An important consideration of our proposal is that the RT difference between SD task-switch and task-repeat saccades reflects a switch-cost and is not related to a planning facilitation for task-repeat trials due to the consecutive presentation of a single trial-type (Wylie and Allport, 2000). In a purpose-designed study addressing this issue, Tari et al. (2019) found that RTs for SD task-repeat saccades and SD saccades performed in a separate block did not reliably differ and were less than SD task-switch trials. In other words, the RT difference between SD task-switch and task-repeat saccades is the result of a switch-cost. Additionally, it could be argued that the increase in RTs for task-switch saccades reflects an implicit – or explicit – control strategy wherein participants increase planning times to improve endpoint accuracy (i.e., speed-accuracy trade-off) (Fitts, 1954). In addressing this issue, we note that results for saccade duration, gain and gain variability did not differ across SD task-switch and task-repeat saccades. In fact, the only difference associated with saccade execution metrics was that MD saccades produced longer durations and increased gain variability than SD saccades – a finding independent of our exercise manipulation. This is an expected finding and one work in non-human primates has shown to relate to the absence of veridical target location at movement onset (i.e., MD saccades) decreasing saccade-related neuron activity in the superior colliculus and rendering slower and more variable responses (Edelman and Goldberg, 2001).

### Experiments 1 and 2: A Post-exercise Reduction in the Unidirectional Switch-Cost

Post-exercise RTs for SD task-switch saccades were reliably longer (8 ms, CI_95__%_ = 7) than their task-repeat counterparts, whereas MD task-switch and task-repeat saccades did not differ and were within an equivalence boundary. Thus, a unidirectional switch-cost was observed at the post-exercise assessment. Notably, however, Figure 2C shows that the *magnitude* of the switch-cost was reduced post-exercise and was independent of any pre- to post-exercise changes in saccade duration, gain or gain variability. Of course, on its own the pre- to post-exercise change in the magnitude of the switch-cost could be attributed to a practice-related improvement in our oculomotor task. For that reason, Experiment 2 involved a non-exercise control condition and produced a pre- (28 ms, CI_95__%_ = 10) and post-break (26 ms, CI_95__%_ = 12) unidirectional switch-cost that did not reliably differ and were within an equivalence boundary to Experiment 1’s pre-exercise switch-cost (28 ms, CI_95__%_ = 9). Accordingly, the combined findings of Experiment 1 and 2 support the assertion that 20-min of aerobic activity improved task-switching efficiency and is a result we interpret to reflect an exercise-based improvement to cognitive flexibility.

At least two issues require addressing. The first is why the current task-switching paradigm indicated a post-exercise improvement in cognitive flexibility, whereas previous work has provided equivocal support. One explanation is that fitness level mediates a post-exercise executive function benefit given Tomporowski and Ganio’s (2006) report that recreationally active participants did not demonstrate an exercise-specific benefit to cognitive flexibility, and given Tsai et al.’s (2016) report that high-fit – but not low-fit – participants elicit a post-exercise benefit in cognitive flexibility. Moreover, Themanson et al. (2006) reported a “…relatively smaller global switch cost” (p. 1335) on a parity judgment task for older adults (i.e., >59 years of age) classified as high- versus low-fit; however, it is important to note that this study involved a chronic exercise manipulation based on participants’ self-report of kilocalories of energy expended. We, however, do not believe that fitness level represents a parsimonious explanation in light of the present study’s observation of an exercise-specific benefit to cognitive flexibility in a corpus of participants with a similar level of recreational fitness as Tomporowski and Ganio (2006). Further, a recent purpose-designed meta-analysis reported that a single bout of exercise benefits executive function (as primarily assessed with inhibitory control and working memory tasks) across the continuum of low- to high-fitness (Ludyga et al., 2016). An alternate explanation is that task-switching paradigms involving non-executive components (i.e., numerosity, parity and consonant/vowel judgments) used in previous studies precluded the ability to reliably detect subtle post-exercise improvements in task-switching. This explanation is consistent with findings showing that a single-bout of exercise does not influence simple or choice RT (Fleury and Bard, 1987; McMorris and Keen, 1994; Aks, 1998), visuo-perceptual recognition (Bard and Fleury, 1978) or numerical processing (Zervas, 1990). Further, and to our knowledge, no studies have addressed whether a single bout of exercise directly improves language or parity judgments in healthy young adults (see Table 1 of Ludyga et al., 2016); however, such a benefit would be at odds with evidence reporting that single bout (Lambourne and Tomporowski, 2010; Chang et al., 2012) and chronic (Colcombe and Kramer, 2003; Hillman et al., 2008) exercise provide the most reliable benefit to executive function.

The second issue is a putative mechanism(s) for the exercise-based improvement in task-switching efficiency. Some neuroimaging (i.e., fMRI) studies have reported an improvement in task-dependent activity within parietal and frontal regions post-exercise (e.g., Li et al., 2014; Chen et al., 2016), whereas other work reported decreased activity within the same frontoparietal networks (e.g., MacIntosh et al., 2014; Chang et al., 2017). Thus, the fMRI literature is equivocal with regard to post-exercise changes in regional activity – a result attributed to between-experiment heterogeneity in exercise durations/intensities and the time frame of post-exercise data capture (Mehren et al., 2019). That being said, we note from the general oculomotor literature that the prefrontal cortex (PFC) is a primary substrate for generating task-switching signals (Rushworth et al., 2002) and that Everling and Johnston (2013) reported that the PFC provides excitatory inputs to the superior colliculus that establish the “task-rules” or “task-set” necessary to implement the non-standard MD saccades used here. Moreover, evidence from functional near-infrared spectroscopy (fNIRS) has shown that a single bout of exercise is associated with increased PFC blood flow and oxygenation (Ainslie et al., 2008) and that such a change may provide the metabolic substrates permitting a more rapid dissipation of the neural activity of a non-standard task-set. That increased blood flow is associated with improved task-switching efficiency is a direct extension of Moore and Cao’s (2008) hemo-neural hypothesis’ assertion that functional hyperemia improves excitatory and inhibitory modulation of the local circuitry supporting information processing. Moreover, and although not mutually independent of the hemo-neural hypothesis, exercise has been shown to alter coherent neural activity associated with resting state functional connectivity. Schmitt et al. (2019) reported that a single bout of light intensity (i.e., <35% of lactate threshold) aerobic exercise improved resting state functional connectivity across frontoparietal networks (see also Kelly et al., 2017). Given that task demands for the SD and MD saccades used here require response mediation via a PFC mechanism (see above) *and* attentional-mediated visuo-motor transformations via parietal circuitry (Zhang and Barash, 2000), it is reasonable to assert that the diminished switch-cost is, in part, linked to improved resting state connectivity within frontoparietal networks.

### Study Limitations

The present work employed young participants classified as recreationally active via the GLETQ. As a result, older adults and those with a reduced (or increased) level of fitness may not demonstrate a comparable post-exercise benefit to cognitive flexibility. A second limitation is that the present work employed only a 20-min session at a moderate intensity. Although previous work by our group has shown a post-exercise benefit in oculomotor inhibitory control for 10- and 20-min sessions of aerobic exercise across a continuum of metabolically sustainable intensities (i.e., moderate [80% of lactate threshold (LT)] to heavy intensity (50% of the difference between LT and VO_2__peak/max_) (Heath et al., 2018; Petrella et al., 2019) it is possible that the distinct neural circuitry associated with cognitive flexibility (Gilbert and Burgess, 2008) may show a distinct dose-response relationship to a single-bout of aerobic exercise. It might be the case that an exercise session less than or greater than the 20-min used here (or different exercise intensities) may not influence task-switching efficiency. Future work should therefore determine whether cognitive flexibility elicits a post-exercise improvement across a broader range of exercise intervals and intensities. A third limitation is that we employed a between-groups design to contrast exercise and control (i.e., no-exercise) manipulations. As such, it could be argued that without a within-group comparison of exercise and non-exercise manipulations it cannot be directly concluded that exercise alone imparts a benefit to executive function. In addressing this issue, we note that a most recent study by our group (Shukla, 2020) employed a within-groups design and showed an exercise-specific benefit to cognitive flexibility across a range of post-exercise time points (i.e., immediate, 30-, 60- and 90-min post-exercise).

## Conclusion

A single bout of moderate intensity exercise improved task-switching efficiency and was a result independent of a practice-related improvement in the oculomotor task used here. Accordingly, our results demonstrate that the SD and MD oculomotor task-switching paradigm provides the resolution necessary to detect post-exercise benefits to cognitive flexibility. Moreover, we believe that the present findings add importantly to the literature insomuch as they provide convergent evidence that a single bout of exercise benefits each core component of executive function.

## Data Availability Statement

The datasets generated for this study are available on request to the corresponding author.

## Ethics Statement

The studies involving human participants were reviewed and approved by Health Sciences Research Ethics Board, University of Western Ontario. The patients/participants provided their written informed consent to participate in this study.

## Author Contributions

DS and MH conceptualized the study, contributed to the methodology, and investigated the study. MH wrote the original draft of the manuscript, acquised the funding, contributed to the resources, and supervised the study. DS wrote, reviewed, and edited the final version of the manuscript.

## Conflict of Interest

The authors declare that the research was conducted in the absence of any commercial or financial relationships that could be construed as a potential conflict of interest.

## Footnotes

1 An interesting feature of Li et al.’s (2014) study is that n-back behavioral performance did not vary from pre- to post-exercise assessments. In a similar vein, Hillman et al. (2003) reported that post-exercise Ericksen flanker task electroencephalography revealed modulation of the P3 event-related brain potential; however, behavioral performance (as measured via RT) did not reliably vary from pre- to post-exercise. In spite of the absence of a post-exercise behavioral effect, Hillman et al. (2003) concluded that aerobic exercise benefits executive control mechanisms.

2 Some studies have contrasted task-switching in adult regular exercisers and non-exercisers (Hillman et al., 2006; Themanson et al., 2006) and provide some evidence that regular exercisers – and those with increased fitness – have improved task-switching efficiency. In contrast, work involving children has demonstrated limited evidence that regular exercise influences task-switching efficiency (for review, see Guiney and Machado, 2013). Importantly, the above research employed current fitness status and/or a long-term exercise intervention, whereas the current work focused on whether a single bout of exercise influences task-switching.
